# Minerals: An Untapped Remedy for Autoimmune Hypothyroidism?

**DOI:** 10.7759/cureus.11008

**Published:** 2020-10-17

**Authors:** Seyad Zulficar Ali Khan, Rayan M Lungba, Uvie Ajibawo-Aganbi, Swathi Veliginti, Maria V Perez Bastidas, Sania Saleem, Ivan Cancarevic

**Affiliations:** 1 Research and Development, California Institute of Behavioral Neurosciences & Psychology, Fairfield, USA; 2 Primary Care & Emergency, Ministry of Health Oman, Salalah, OMN; 3 Internal Medicine, California Institute of Behavioral Neurosciences & Psychology, Fairfield, USA

**Keywords:** essential nutrients, autoimmune thyroiditis, hashimoto's thyroiditis, hypothyroidism, nutraceuticals, zinc, selenium, magnesium, iodine

## Abstract

For decades, the focus of managing autoimmune hypothyroidism has been on thyroxine replacement. Correcting lab parameters such as thyroid stimulating hormone (TSH) has been a primary goal. The increasing prevalence of Hashimoto’s thyroiditis (HT) continues to impact the quality of life in patients. We believe a holistic approach to this disease entity, considering its underlying complex etiopathogenesis, would benefit patients. Nutraceuticals are combinations of essential nutrients and are becoming a part of novel medical treatments despite the lack of regulation. This review aims to present a concise summary of recent developments regarding minerals such as zinc, selenium, magnesium, iron, and their potential clinical benefit as nutraceuticals in patients with HT. We have explored the potential benefits and associations of these minerals in HT and thyroid function. We reviewed relevant articles, metanalyses, and clinical trials in literature platforms such as PubMed, PubMed Central, and Google Scholar. Significant data found in the literature suggesting a potential health benefit of these minerals in HT though there were many studies to the contrary. Many trials demonstrated the restoration of residual symptoms, reversal of HT-associated thyroid morphological changes, and improvement in thyroid functions. Many of these trials lack statistical power due to the small sample sizes, and their external validity may be questionable due to unaccounted confounding factors. In our opinion, to support an evidence-based holistic clinical approach, further scientific evidence is needed. The association of these elements in thyroid function necessitates more large scale pragmatic trials to elucidate the benefits of nutraceuticals in HT.

## Introduction and background

Thyroid autoimmunity, commonly mentioned as autoimmune thyroid diseases (AITD) in literature, encompasses various disease entities that arise due to our immune system being intolerant to specific thyroid antigens. This immune intolerance can trigger a cascade of cellular and humoral immune reactions that could eventually damage the thyroid cellular infrastructure [1]. Immune-mediated insults often result in the dysfunction of this vital gland. As one of the most affected organs in the body by autoimmunity, researchers are puzzled by the low immune tolerance of this organ. Researchers have explored the possible interplay of environmental, nutritional, and genetic factors triggering this autoimmunity. The most prevalent disease entities resulting from autoimmune insult are Hashimoto’s thyroiditis (HT) and Grave’s disease (GD) [1]. HT manifests commonly as hypofunctioning of the gland and often reflects clinically with hypothyroidism features such as fatigue, constipation, irregular menses, cold intolerance, and weight gain, to name a few [1,2]. Meanwhile, GD manifests clinically with hyperthyroidism features such as palpitations, anxiety, menstrual dysfunction, heat intolerance, and weight loss [1,2]. 

HT is the most common cause of hypothyroidism in the developed world and areas with sufficient iodine repletion. In contrast, iodine deficiency still is the number one cause for hypothyroidism in regions where nutritional iodine deficiency exists [2]. HT is more commonly associated with anti-thyroid peroxidase antibody (TPO-Ab) and anti-thyroglobulin (TG) antibodies in serum resulting in lymphocyte infiltration, fibrosis in later stages. Its prevalence has been around five for every 1000 residents in the USA. HT more commonly affects females, and the average age of onset is 35-45 [3]. The prevalence is in an upswing bringing the spotlight on iodine overcorrection as a possible causal factor and necessitating further research on other influences in play. Analysis in Denmark indicated a 55% co-occurrence rate of AITD in monozygotic twins compared to 3% in dizygotic. The study also suggested that around 79% of predisposition can be attributable to genetic factors and the remaining 21% to environmental, nutritional, and other influences [4].

In recent years, the role of nutritional deficiencies in the pathogenesis, disease perpetuation, and clinical manifestations of HT has drawn much attention. For instance, vitamin D deficiency and excessive iodine intake could potentially be the underlying cause of HT development [5,6]. There is evidence of a positive relationship between vitamin D and thyroid stimulating hormone (TSH), thyroxine (T4), and triiodothyronine (T3) levels in literature, yet unresolved questions remain. In one study, vitamin D was deficient in 76.7% of HT patients and 70% of Graves’ disease patients compared to 20.0% in healthy people. However, its role in preventive and therapeutic aspects was not proven [5]. Iodine overcorrection is known to increase reactive oxygen species (ROS) in the thyroid gland. Accumulation of ROS can activate inflammatory pathways leading to thyrocyte apoptosis, thus playing a pivotal role in HT [6].

Apart from vitamin D and iodine excess, the crucial role of zinc, selenium, iron, and magnesium in thyroid pathophysiology has been a subject of interest. A small subset of 10 to 15% of the population with clinical manifestations of HT is, in fact, negative for antibody titers. In them, positive antibody titers only signified the symptom severity [7]. Further supporting the complexity of HT, studies reveal up to 10% of hypothyroid patients continue to experience symptoms even after receiving treatment to normalize serum TSH levels [8]. Possible explanations for this could be individual differences in sensitivity, unique variations in average values, and possibly an interplay of nutritional and environmental influences.

**Figure 1:**
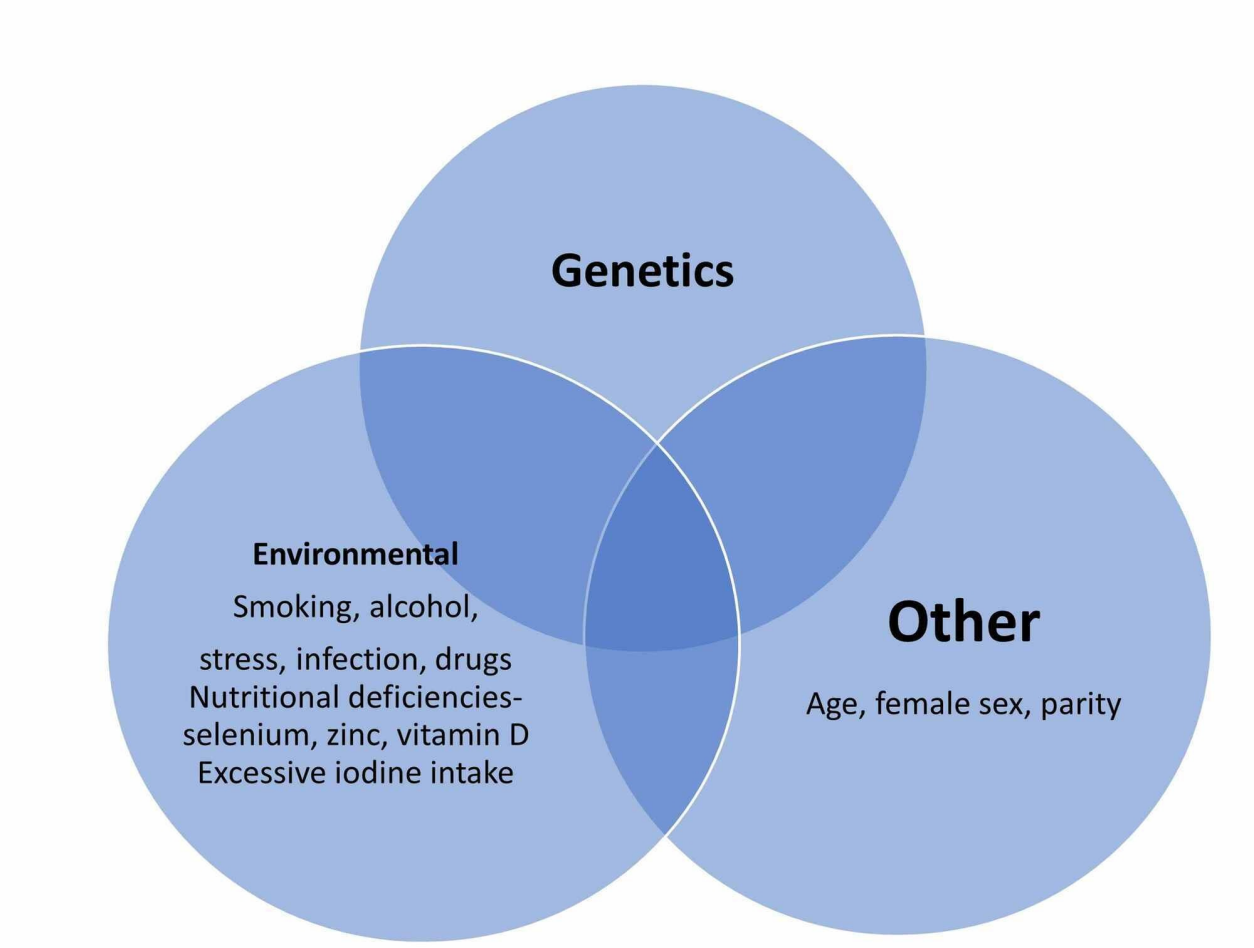
Factors influencing the development of Hashimoto's thyroiditis

HT is presently managed with daily thyroxine supplementation to overcome the hypothyroid symptoms. Established protocols help clinicians decide on the appropriate initial dosing and then titer it to bring TSH within the standard reference range [3]. This review article will focus on how undetected nutritional deficiencies of zinc, selenium, iron, and magnesium can adversely impact patients with HT. Importantly we will search for any added benefit in addressing these deficiencies in HT patients in their overall quality of life, disease control, and lab parameters. We will explore the literature in PubMed, PubMed Central, and Google Scholar to enhance our knowledge of recent developments in this front. Our search will include both animal-model studies and human clinical trials to understand the associations better. We aim to determine if there is compelling evidence in the literature to incorporate screening for mineral deficiencies and supplement the needed minerals as part of comprehensive HT management. Figure 1 represents the various factors involved in the development of autoimmune thyroiditis [2,5-7].

## Review

Role of apoptosis in autoimmune thyroiditis

Thyroid autoimmunity is an organ-specific immune disorder caused by aberrant immune regulation. Underlying pathogenesis can involve two distinct mechanisms in Grave’s disease and Hashimoto’s thyroiditis [9]. Researchers once thought that antibody-mediated cellular destruction and T-cell mediated cytotoxicity are the primary underlying mechanisms of HT. Recently, however, researchers have highlighted the crucial role of apoptosis in HT propagation [9]. Multiple in vitro studies have revealed high expression of pro-apoptotic molecules such as Fas, FasL, and Bax in thyroid follicular cells of HT patients, and reduced expression of the same in invading lymphocytes. This finding indicates an increased propensity for thyrocyte apoptosis and lymphocytes’ survival. In contrast, studies done in GD show reduced expression of pro-apoptotic molecules and increased anti-apoptotic Bcl-2 expression in thyroid cells, which supports thyroid cell survival [10].

What triggers the cascade of apoptosis in thyroid glands of HT patients is yet poorly understood. A reasonable explanation would be an environmental, viral, nutritional deficiency, and oxidative stress on genetically susceptible patients [7]. For instance, essential microelements such as zinc, selenium, and magnesium all play a pivotal role in preserving and regulating cellular oxidative state [11-13]. Their role in antioxidant defense help prevents cell damage from oxidative stress [11-13]. Any imbalance in the redox state and defect in the antioxidant defense mechanism can trigger apoptosis and inflammation. Thus, the oxidative stress of thyroid cells is an essential factor in HT propagation [14].

Oxidative stress as an underlying factor in Hashimoto's thyroiditis

The significance of oxidative stress (OS) in the pathophysiology of HT has drawn much attention in recent years [14]. Reduction and oxidation (redox) are critical metabolic steps fundamental to cellular homeostasis and metabolism. They play a crucial role in cellular defense against invading infectious agents and signal transductions in immune processes [14]. Any imbalance in the redox state can result in elevated concentrations of oxidants such as reactive oxygen species (ROS), reactive nitrogen species (RNS), reactive sulfides (RS) in cells [14,15]. This increase in oxidants relative to reductants causes oxidative stress to cell components [14,15]. Increased OS destabilizes the cell membrane and other vital cell structures, causing alterations in DNA and its repair and degradation [15].

Antioxidants counteract oxidative stress. Some essential antioxidants are enzymatic such as catalases, glutathione peroxidases, superoxide dismutase, and thioredoxin system [16]. Some are non-enzymatic such as vitamins A, C, and E [16]. Recent studies have unraveled the potential role of ROS in the induction of autoimmunity and apoptosis [16,17]. ROS accumulation cause elevated oxidative stress, DNA instability, disruption of DNA repair, and degradation mechanisms [17]. This disruptive role of ROS results in the accumulation of single-stranded DNA (ssDNA) and double-stranded DNA (dsDNA) fragments in the cytoplasm [16,17]. Both ssDNA and dsDNA fragments are potent immune stimulators; they do this by inducing interferon (IFN) genes to increase the production of IFN [17]. Interferon, in turn, triggers autoreactivity and inflammation. The defective DNA repair mechanism is also a cause of innate immunity activation and inflammation [17].

**Figure 2:**
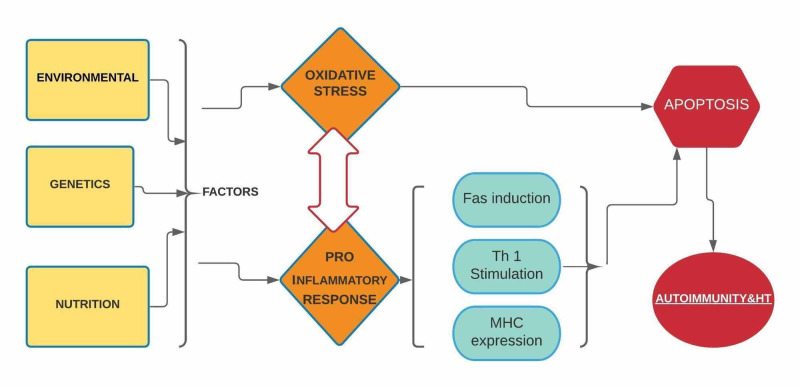
Pathway to autoimmunity & Hashimoto's thyroiditis MHC: major histocompatibility complex, Fas ligand: a transmembrane protein

A study highlighted the crucial role of OS in HT by demonstrating a significant negative correlation of anti-TPO antibody with antioxidant status. They also found a similar negative correlation between antioxidant thiol and anti-TG antibody [14]. Baser et al., in a similar study, demonstrated a positive correlation of oxidative stress with the anti-TG antibody [18]. Ruggeri et al., in his research, suggested that the TPO antibody can be a marker and predictor of oxidative stress [19]. In an animal model study, ROS accumulation in thyroid cells promoted increased fragmentation of thyroglobulin, unmasking potential epitopes to innate immunity [20]. A similar study in mice revealed that increased oxidative stress caused increased susceptibility to iodine-induced apoptosis of thyrocytes [21]. Figure 2 highlights the progression to HT [10,14,17].

Selenium, a crucial element in thyroid function

Since its discovery, research has continued to unravel the vital role of selenium in the human body. Once side-lined as a carcinogen, discoveries of its diverse role in human health has perplexed researchers. It promotes cardiovascular wellbeing, endocrine regulation, and plays a more significant role in immunity. Selenium directly incorporates as the 21st amino acid selenocysteine in various proteins. Interestingly the highest concentration of selenium is also found in the thyroid. Three out of 35 known selenoproteins play a crucial role in thyroid function. Some selenoproteins implicated in thyroid function are iodothyronine deiodinase-1 (DIO1), iodothyronine deiodinase-2 (DIO2), which are essential to maintaining intracellular and systemic active thyroid hormone (T3) levels [13]. Iodothyronine deiodinase-3 (DIO3), the other selenoprotein, helps to inactivate excess thyroxin [13,22]. Selenoprotein K (selenok) also plays a vital role in calcium influx in immune cells [13,22]. Some selenoproteins such as glutathione peroxidase, thioredoxin reductase, and sulphoxide reductase play a crucial role in immune regulation [22]. Glutathione peroxidase (GP)1, GPX3, and GPX4 catalyze peroxides and protect thyrocytes from oxidative damage [13]. It is envisaged that selenium is vital to maintain redox homeostasis, for efficient DNA damage repair, and to regulate proinflammatory cytokine synthesis. It also has a direct effect on innate immunity through the regulation of macrophage function [22].

Many studies have revealed an association of selenium deficiency with thyroid autoimmunity. A metanalysis published in 2016 explored the benefit of selenium supplementation in HT. They concluded that selenium supplementation could significantly reduce thyroid autoantibodies in patients with HT. They compared both patients on thyroxin substitution and patients not on thyroxine substitution. Their study revealed the beneficial effects of three-month selenium supplementation in patients not on thyroxine therapy [23]. Pirola et al. conducted a prospective clinical trial to evaluate the benefit of 83mcg once-daily selenium supplementation for four months in patients with HT. The trial demonstrated the restoration of euthyroidism in 31.3% of responders amongst the selenium group compared to only 3.1% in controls [24]. In a separate study, Pirola et al. demonstrated the TSH lowering effect of short-course selenium supplementation. His research also suggested that the TSH lowering effect lasted six months after the withdrawal of selenium supplements [25]. In an earlier study conducted by Gartner and co-workers, they showed a decrease of 40% in mean TPO antibody titers after supplementing selenium. They backed their study with ultrasound imaging of the thyroid gland, which showed a reduced echogenicity in the selenium group compared to the controls, indicating a beneficial effect in lowering thyroid inflammation in patients with HT [26].

A prospective randomized clinical trial conducted in Italy in 2017 enrolled 168 patients with HT to evaluate the added benefit of Myo-inositol and selenium co-supplementation. After six-months of supplements, improvements in subjective symptoms and thyroid function parameters were assessed. The study concluded that 83mcg of selenium supplementation with 600mg of inositol daily significantly improved their symptoms and restored the euthyroid state [27].

Pregnancy increases the basal metabolic rate, and the demand for thyroxine rises during pregnancy. A study named SERENA demonstrated the benefit of selenium as a supplement in pregnant women with HT. The study enrolled 45 pregnant women with AITD and started daily selenium supplementation of 83mcg, and assessments at the first-trimester end, at term, and postpartum were done. Although the study failed to show any added benefit in quality of life and pregnancy-related complications, it revealed significant reductions in TG antibody and TPO antibody titers (P<0.01) [28]. A study in the UK explored the benefit of low-dose selenium supplements in thyroid antibody titers of pregnant women from low iodine intake communities. The study classified patients based on their thyroid antibody status. It concluded that significant TSH reductions only in the TPO-Ab group, and TPO-Ab titers did not change significantly. Considering that study did not recruit patients with known thyroid diseases, it is unclear how patients with HT and on thyroxine supplements would have responded to low-dose selenium supplements. This study also raises the possibility of iodine status as a potential confounding factor in studies related to thyroid and selenium intake [29].

Our search retrieved a total of 35 relevant clinical trials and three metanalyses. Two studies assessed the co-supplementation of selenium and myoinositol in AITD [27]. One study assessed selenium and vitamin C co-supplementation in AITD, and three other studies assessed selenium supplementation in thyroid autoimmunity of pregnant women [28,29]. There is also one study investigating selenium and vitamin D co-supplementation in AITD. There are 11 other clinical trials related to the assessment of selenium supplementation on thyroid function in healthy people. There are 16 clinical trials directly assessing the effect of selenium supplementation in AITD. Many of these trials evaluated the effect of selenium supplementation on TPO antibodies. Many studies have found a significant decrease in TPO-Ab after three to 12 months of selenium supplementation [30,31]. However, a few studies did not find a substantial outcome in antibody titers [32]. One study assessed the effect on the quality of life though it failed to prove any significant outcome [33].

In our assessment, the above studies, in general, need to be seen in the light of limitations such as small sample sizes and their potential for confounding factors like age, gender, thyroid status, and other possible comorbidities. Though these studies are promising, in the future, we suggest extensive population-based pragmatic studies that would help delineate the beneficial role of selenium supplementation in clinical practice. One such community-based, large pragmatic study named CATALYST (chronic autoimmune thyroiditis quality of life selenium trial) specifically looks into the outcome in quality of life in AITD, and results are awaited [34]. Table 1 summarizes some of the important studies related to selenium supplementation in AITD. 

**Table 1:** Influence of selenium in AITD. OD: once daily, AITD: autoimmune thyroid disease, QoL: quality of life, RCT: randomized control trials, TPOAb: anti-thyroid peroxidase antibody, TgAb: anti-thyroglobulin antibodies, T4: thyroxine, T3: triiodothyronine

Author & Year	Aim of selenium supplementation	Study type & (n)	Supplementation method	Results
Pirola et al. 2016 [24]	Effect on subclinical hypothyroidism in AITD	RCT, n-196	83mcg-OD for 4-Months	17.2% achieved euthyroidism.
Pirola et al. 2019 [25]	Effect on TSH, autoimmune markers in AITD	Prospective observational. n-50	83mcg-OD for 4 Months	48.9% of responders achieved euthyroidism. No significant change in TPOAb & chemokines.
Gartner et al. 2002 [26]	Effect on antibody titers, thyroid function, volume & QoL in AITD.	RCT, n-70	200mcg-OD for 3-Months	40% reduction of TPOAb in the selenium group compared to 10% in placebo. No significant changes in TgAb, T4, T3. USS improved in nine patients.
Mantovani et al. 2019 [28]	Effect on AITD among pregnant women.	RCT, n-45	83mcg-OD Till 6-Months postpartum.	Significant reductions in TPOAb (P<0.01), TgAb (P<0.01). No changes in QoL.
Kyrgios et al. 2018 [31]	Effect of high dose selenium on AITD	RCT, n-71	200mcg- OD for 6-Months	Reductions in TgAb (P=0.021). TPOAb not significant (P=0.219). USS no changes.
Bonfig et al. 2010 [32]	Effect on antibody titers	RCT, n-49	Group A-Thyroxine alone.Group B-Thyroxine + selenium 100mcg.Group c-Thyroxine + selenium 200mcg	Reductions in TgAb in Group A (P=0.03) & Group C (P=0.01). Group B, not significant (P=0.06). No significant changes in TPOAb in all groups.
Eskes et al. 2013 [33]	Effect on euthyroid patients with positive TPOAb.	RCT, n-61	200mcg- OD for 6-Months	No effect on QoL and TPOAb titers.

Zinc as an antioxidant

We cannot overstate the vital role of zinc in the human body. It is a potent antioxidant, anti-inflammatory, and immune regulating element [35]. Its deficiency increases autoimmune susceptibility in general [36].

A randomized control trial conducted in 2015 explored the potential benefit of both selenium and zinc either alone or in combination with thyroid function. The study concluded a beneficial outcome with either zinc alone or in combination with selenium in thyroid function [37]. Another study aimed to evaluate the role of zinc supplementation in hypothyroidism in patients with Down’s syndrome. The study demonstrated improved thyroid parameters, specifically TSH, after supplementing zinc for six months [38]. In an earlier study conducted in 2009, researchers explored the benefit of zinc supplementation in male and female patients with goiters. The study reported a beneficial outcome in thyroid function after six months of zinc supplementation [36]. Though the above studies indicate a potential benefit in thyroid status with zinc supplementation, their role in autoimmune thyroiditis is yet to be explored and established. Previous research on zinc’s role in multiple autoimmune diseases suggests a potential benefit in preserving immune tolerance [39].

Magnesium for iodine uptake

To date, limited data available in the literature on the association of magnesium in thyroid function. However, studies on the benefit of magnesium supplementation in the body’s overall oxidative status and inflammation have been published [11,40]. Thyroid hormones play a vital role in mitochondrial function. Adenosine triphosphate (ATP) production in mitochondria is essential to maintain energy-dependent cell reactions, including iodine uptake through sodium-iodine symporters, a crucial step in thyroid hormone production. Magnesium is an essential element in oxidative phosphorylation as part of complex V [40]. A study concluded that elevated thyroid vasculature associated with HT’s inflammation correlated negatively with serum magnesium levels [41]. Another similar study analyzed the outcome of co-supplementation of magnesium, selenium, and coenzyme Q10 in patients with benign thyroid conditions. After two to four years of supplementation, they found a significant drop in antibody titers and normalization of thyroid morphology as evidenced by high-resolution USS [42]. This study certainly has limitations considering the small sample size and potential for biases and confounding factors.

HT encompasses a diverse range of symptoms, which can even present in patients with unremarkable lab and physical parameters. Symptoms such a chronic fatigue, irritability, low concentration, anxiety, nervousness, and even unexplained miscarriages pose a dilemma to medical professionals. Commonly these symptoms are categorized as psychosomatic or medically unexplained symptoms (MUS) [43]. Not recognizing or failing to address these symptoms can adversely affect the quality of life in these patients. There is ample evidence in the literature of co-existing physical and psychosocial stressors in HT and hypothyroidism [43]. A study found that hypothyroidism and other physical/psychosocial stressors can increase magnesium demand. This study included 166 euthyroid and 108 hypothyroid patients. It assessed the stress levels by a scoring system before and after magnesium supplementation in three and six months. They found that 90% of patients had their psychosomatic symptoms resolved after magnesium supplementation. A significant portion of patients also reported improved quality of life [44]. Though these studies may not hold sufficient evidence to suggest magnesium’s benefit in hypothyroidism scientifically, they highlight the need for future studies in this direction.

Iron for optimal thyroid functioning

Thyroid peroxidase (TPO), a hemoprotein in thyrocytes, has a significant role in thyroid hormone synthesis. It is crucial for iodine oxidation and integration into thyroglobulin. There is evidence from multiple animal model studies that TPO activity diminishes under iron deficiency. These studies imply the need for iron sufficiency to maintain optimum thyroid function [45]. Further, Hu et al., in his studies in rats, demonstrated iron deficiency alone without anemia can lead to suboptimal thyroxine production [46].

In his study, Yu et al. concluded that iron deficiency could be an independent risk factor for hypothyroxinaemia in pregnant and non-pregnant mothers [47]. In his study in pregnant women, Zimmermann et al. demonstrated that reduced iron stores could negatively correlate with the TSH while showing a positive relationship between iron stores and thyroid hormones. He concluded that optimal iron stores could achieve a better thyroid function [48].

A large cohort study demonstrated the significance of thyroid hormone in maintaining hemoglobin and hematocrit levels. The study reveals TSH correlated negatively with iron stores, transferrin saturation [49]. Thyroid hormones augment erythropoietin gene expression in the kidney and induce hematopoiesis. Hypothyroid status reduces basal metabolic rate resulting in decreased demand for oxygen and erythropoiesis. So, reduced thyroid hormones do lower erythropoiesis, further signifying the importance of sufficient iron stores [49].

**Figure 3:**
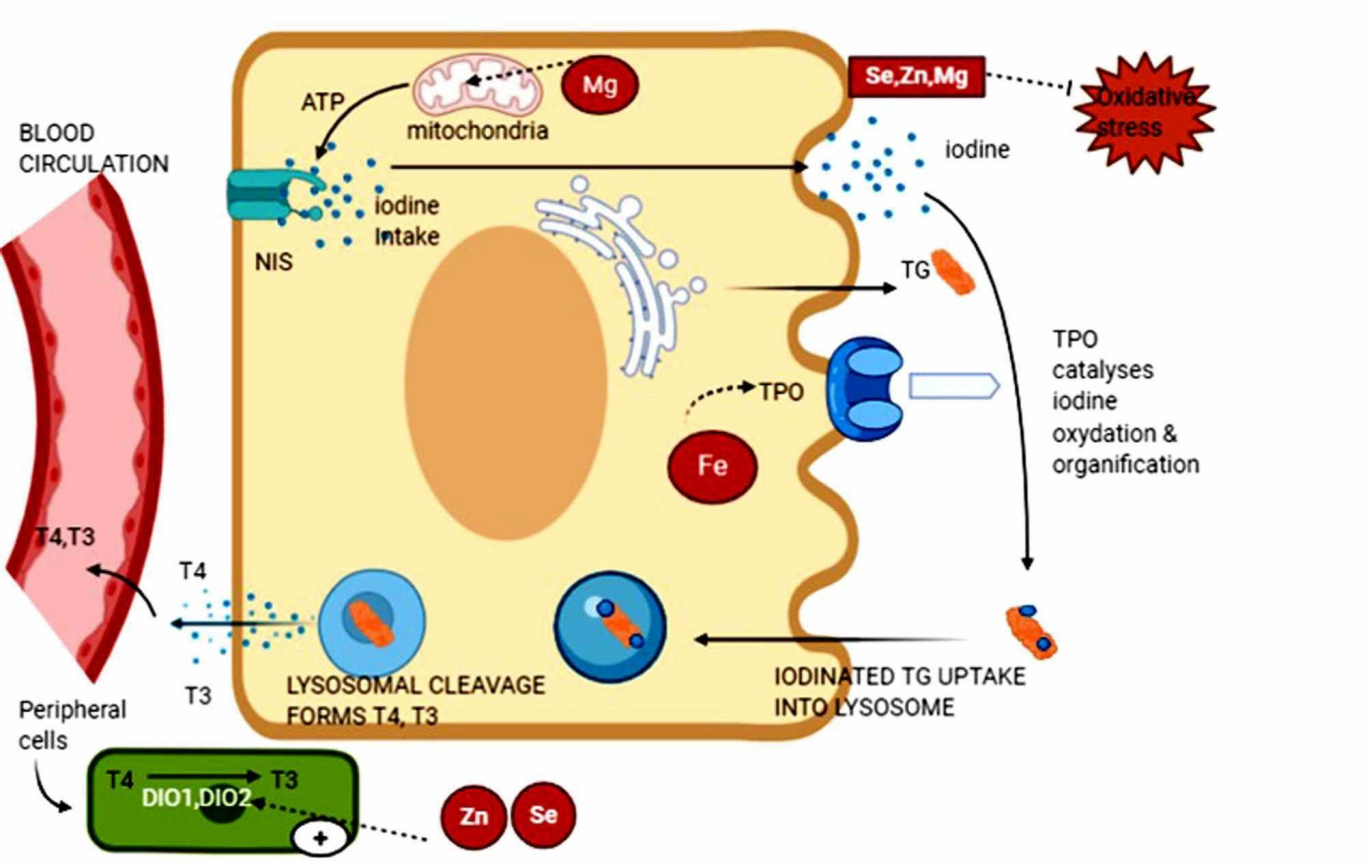
Overview of the influence of minerals in thyroid physiology NIS: sodium iodide symporter, TPO: thyroid peroxidase enzyme, DIO1,2: iodothyronine deiodinase 1,2, ATP: adenosine triphosphate, TG: thyroglobulin, T4: thyroxine, T3: triiodothyronine

Further, AITD is associated with multiple other autoimmune diseases that could be an additional causal factor for anemia [50]. These include pernicious anemia, celiac disease, autoimmune hemolytic disease, and rheumatism, which causes anemia of chronic diseases. In women, heavy menstrual bleeding is a frequent manifestation of hypothyroid status and often results in iron deficiency [50]. Data on the prevalence of anemia in subclinical thyroid dysfunction are inconsistent in the literature. Studies have revealed the incidence of anemia among hypothyroid patients at 57% [50]. The importance of maintaining iron sufficiency in hypothyroid patients can be attributed to its role in thyroid hormone synthesis and due to the increased susceptibility of developing anemia in these patients. Further large-scale cohort studies are needed to determine if any causal relationship exists between iron deficiency and AITD. Figure 3 demonstrates the influences of these minerals in thyroid function [11,22,35,40,45].

Limitations in our review

Our review has some limitations; we did not carry out quality assessments on the chosen studies and articles. Some of the researches related to etiopathogenesis are based on animal models. Most of the clinical trials were with inadequate statistical power and non-pragmatic. They may not apply to different populations and settings. The decision to supplement nutraceuticals should not be based solely on hypothetical health benefits. Dosing requirements can vary based on individual diet practices and geographical areas due to variations in soil nutrients and agricultural practices. Potential for harm due to high doses, especially in susceptible populations such as children and the elderly, also needs to be considered.

## Conclusions

In this review, we have attempted to present a concise summary of the potential benefits of micronutrients zinc, selenium, magnesium, and iron on overall thyroid function and in HT. Oxidative stress and apoptosis play a significant role in HT’s etiopathogenesis. Oxidative DNA damage and cell apoptosis are initial events triggering autoimmunity in the thyroid. Preserving oxidative status and optimizing nutrients can help improve thyroid function. It is evident from many studies that selenium and zinc play an essential role in preserving thyrocytes’ oxidative status and prevent apoptosis and cell damage. Magnesium has a critical role in the functioning of sodium iodide symporter, which helps maintain a steady iodine supply for thyroid hormone synthesis. Iron deficiency is strongly associated with hypothyroid status, and sufficient iron stores are vital for the thyroid function. Large scale clinical trials are needed in the future to consider incorporating supplements as a novel evidence-based remedy in clinical practice.
